# Effect of Addition of Spheroidal Cellulose Powders on Physicochemical and Functional Properties of Cosmetic Emulsions

**DOI:** 10.3390/polym17141926

**Published:** 2025-07-12

**Authors:** Emilia Klimaszewska, Marta Ogorzałek, Małgorzata Okulska-Bożek, Ewa Jabłońska, Hanna Wyłup, Zofia Nizioł-Łukaszewska, Ryszard Tomasiuk

**Affiliations:** 1 Department of Cosmetology, Faculty of Medical Sciences and Health Sciences, Casimir Pulaski University of Radom, Chrobrego 27, 26-600 Radom, Poland; m.ogorzalek@urad.edu.pl (M.O.); m.okulska@urad.edu.pl (M.O.-B.); e.jablonska@urad.edu.pl (E.J.);; 2 Department of Technology of Cosmetic and Pharmaceutical Products, Medical College, University of Information Technology and Management in Rzeszow, Sucharskiego 2, 35-225 Rzeszow, Poland; zniziol@wsiz.edu.pl; 3 Department of Basic Medical Sciences, Faculty of Medical Sciences and Health Sciences, Casimir Pulaski University of Radom, Chrobrego 27, 26-600 Radom, Poland; r.tomasiuk@urad.edu.pl

**Keywords:** modified natural polymers, cellulose powder, cosmetic emulsions, Turbiscan stability index, dynamic viscosity, texture analysis, degree of skin hydration, sensory analysis

## Abstract

The purpose of this study was to demonstrate the feasibility of using spheroidal cellulose powders with different particle sizes (2 and 7 µm) in face creams and to evaluate their effect on selected physicochemical and performance properties of these products. A series of prototypes of facial creams with spheroidal cellulose were prepared. The following tests were carried out: stability, dynamic viscosity, texture analysis, degree of skin hydration, and evaluation of sensory appeal by consumers. It was observed that none of the creams showed instability over time. The addition of powdered spheroidal cellulose was found to increase dynamic viscosity and hardness and reduce the adhesion strength of the tested emulsions to the base face cream. A positive effect of the presence of polymeric raw materials on the level of skin hydration was observed. The most favorable results were obtained for the E4 cream prototype containing spheroidal powders of both 2 and 7 µm particle size at a weight ratio of 2.5 to 2.5. In addition, according to the members of the sensory panel, the E4 face cream was best evaluated and showed sensory benefits. The study concluded that spheroidal cellulose powders are a promising biodegradable alternative to microplastics in cosmetics.

## 1. Introduction

Plastic waste is growing rapidly worldwide due to the widespread use of polymeric materials in many areas of life. These wastes usually do not decompose. Their presence in the ecosystem poses a serious threat to both humanity and the animal and plant world. The observed environmental pollution from plastic waste poses a problem with numerous production management challenges, limiting the achievement of sustainable development objectives [1,2,3]. Therefore, new, environmentally safe solutions are constantly being sought. This aspect also applies to the cosmetics industry. Microplastics, such as polyethylene (PE), polypropylene (PP), polyethylene terephthalate (PET), and polyamides (PA), are widely used in the cosmetics industry. Primary microplastics, as opposed to secondary microplastics formed by mechanical abrasion and wear of large plastic products, are intentionally obtained fine particles of polymeric materials. They are used as multifunctional additives in cosmetic products such as toothpastes, exfoliants, soaps, shower gels, creams, etc. They are used as texture-forming, film-forming, filling, or exfoliating substances [4,5,6]. Unfortunately, they are a significant source of marine and ocean pollution, and they can take up to hundreds of years to decompose in the environment. The difficulty in identifying and disposing of microplastics and plastics less than 5 mm in diameter is a serious environmental problem [4,5,6].

**Figure 1:**
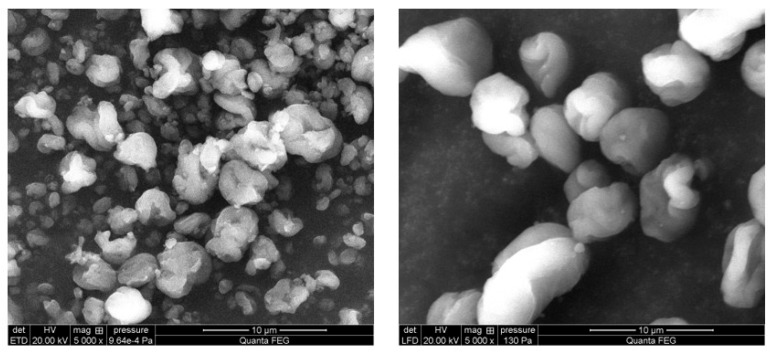
Photo of spheroidal cellulose powders taken with a scanning electron microscope, magnification 5000×, Cellulose 2 µm (left), Cellulose 7 µm (right). FEI Quanta 250 FEG scanning electron microscope (SEM) (FEI Inc., Eindhoven, The Netherlands). Source: own study.

An alternative to synthetic microplastics in cosmetics can be the use of high-quality carboxyl cellulose nanocrystals, processed into spheroidal micron-sized cellulose powder. These raw materials are extracted from a tree called black spruce, which comes from Canadian forests. Their obtaining is in accordance with the principles of sustainable forestry. The trees that were cut down were replaced with others. They are vegan, 100% natural, and biodegradable [7]. Cellulose nanocrystals (CNC) are a modern polymeric material of natural origin. This fact alone is enough to consider them an interesting component of cosmetic formulations. They are obtained using innovative patented technologies from many plants and agricultural residues, such as bamboo [8], jute fibers [9], and potato peels [10]. The physical and chemical properties of nanocrystalline cellulose are influenced by the plant raw material from which it is extracted and the method used to isolate the nanocrystals. Accordingly, cellulose nanocrystals take on different shapes (needles, nanofibers, spheroidal structures) and sizes, which largely determine the possibilities and directions of their application. One of the world’s producers of this material is Canada, and the black spruce (*Picea mariana*) found in Canadian forests is in turn one of the plants from which nanocellulose can be produced. In addition, the use of spheroidal cellulose powders in cosmetics can not only reduce the adverse effects on the aquatic environment but also contribute to the functionality of the cosmetic product, improving some of its characteristics, such as stability, spreading, texture, and other sensory properties, or even lead to the elimination of skin defects (such as fine scratches, scars, or wrinkles) and the reduction of pores [7,10,11,12,13]. Recognition of the possibility of improving the functional properties of emulsions provided the impetus for an attempt to develop formulations of facial creams involving spheroidal cellulose powders and to evaluate their physicochemical and functional properties. Two powders were selected for the study, which differed in particle size (2 and 7 µm). Figure 1 shows a photo of spheroidal cellulose powders taken with a scanning electron microscope. Table 1 shows the particle characterization of cellulose. Physicochemical tests, such as stability, dynamic viscosity, texture analysis, and tests to determine the performance against the skin, were performed for the developed face creams: hydration measurement and consumer evaluation of the sensory appeal.

## 2. Materials and Methods

### 2.1. Materials

The following raw materials have been used to make a series of face creams: Cosme-Phytami^®^ Ivy (INCI: *Hedera Helix (Ivy) Leaf Extract*) from Alban Muller International, Croda (Cracow, Poland); Lanette O (INCI: Cetearyl Alcohol) from BASF Personal Care (Monheim, Germany); Olivem 1000 (INCI: Cetearyl Olivate and Sorbitan Olivate) from Ecospa (Warsaw, Poland); Kem BS (INCI: Sodium Benzoate and Potassium Sorbate) from AkemaS.r.l. (Provincia di Rimini, Italy); and Vitamin E (INCI: Tocopheryl Acetate) from Ecospa (Warsaw, Poland). The main raw materials studied were ChromaPur CV 2 (INCI: Cellulose) from Croda Beauty (Cracow, Poland) and ChromaPur CV 7 (INCI: Cellulose) from Croda Beauty (Cracow, Poland). Their characteristics are shown in Figure 1 and Table 1.

### 2.2. Formulations

Formulations were developed (Table 2) for face creams containing 5% *w*/*w* spheroidal cellulose powder of 2 µm (E2) and 7 µm (E3), and a cream containing both spheroidal powders (2 and 7 µm) in a mass ratio of 2.5:2.5 (E4).

Technology: The ingredients of phase I (*Hedera Helix (Ivy) Leaf Extract*, Cetearyl Alcohol, and Cetearyl Olivate and Sorbitan Olivate) were weighed and combined by mixing on a magnetic stirrer and heated in a water bath to 70 °C. The mixture was stirred until all the ingredients were completely dissolved. Then the ingredients of Phase II (Aqua, Cellulose) were weighed out and combined by stirring on a magnetic stirrer and also heated in a water bath to 70 °C. The mixture was stirred until all the ingredients were completely dissolved. The two phases were combined by adding the aqueous phase to the fatty phase. The whole mixture was stirred on a magnetic stirrer for about 10–15 min, until the two phases were combined. It was cooled to a temperature of about 30 °C. Then the ingredients of phase III (Tocopheryl Acetate and preservative Sodium Benzoate and Potassium Sorbate) were added, and the ingredients were mixed thoroughly.

### 2.3. Methods

#### 2.3.1. Stability Analysis

The stability of the developed emulsions was analyzed using Formulaction’s Turbiscan Lab Cooler instrument. It enables the characterization of physicochemical phenomena such as particle migration and particle size change. The instrument’s measuring head, which is a light source along with two synchronized transmission and backscatter sensors, scans the preparation, moving from the bottom to the top of the vial, collecting data every 40 μm over the entire height of the sample (up to 50 mm). The Turbiscan LAB camera software version 2.0.0.19 was used to analyze the test results. The test results were interpreted on the basis of the TSI (Turbiscan Stability Index).

#### 2.3.2. Dynamic Viscosity

Dynamic viscosity testing of face creams was performed at 22 °C using a Brookfield Rheometer type HA DV III Ultra (Brookfield Engineering Laboratories, INC., Middleboro, MA, USA). Dynamic viscosity was assessed using Helipath-type spindles at a spindle speed of 10 RPM. Five independent measurements were taken for each formulation, and then the values obtained were averaged.

#### 2.3.3. Texture Analysis

The textural properties of the formulated face creams were determined using a Brookfield CT3 texture analyzer (Brookfield Engineering Laboratories, INC., Middleboro, MA, USA). The measurements were conducted using a spherical nylon probe, 25.4 mm in diameter (Brookfield Engineering Laboratories, INC., Middleboro, MA, USA), at an immersion depth of 5 mm, a head travel speed of 0.1 mm/s, and a load of 4500 g. The measurements were recorded at a temperature of 22 °C. The results (hardness and adhesive force) were recorded using the Texture Pro CT software. Adhesive force is represented by a peak negative value, but it is reported as a positive value. The results are presented as the arithmetic mean of five measurements.

#### 2.3.4. Skin Hydration

Skin hydration was tested using the Hydro Pen HPA 10 (Courage + Khazaka electronic GmbH, Köln, Germany), which determined the capacitive resistance of the stratum corneum. Measurements were first performed on a designated 2 × 2 cm area of clean skin of the forearm. Then, 0.5 g of cosmetic was applied to the same area of skin, and the measurement was taken after 2 h. Measurements were performed with approximately 5 s breaks between each subsequent measurement. The final results correspond to the mean of five reproducible experiments. Skin hydration was measured in a room with the following environmental conditions: temperature 22 °C and relative humidity 40–60%. The test subjects (10 women aged 25–45) were acclimatized for 30 min so that blood circulation could return to normal after any physical exercise. All participants gave their informed consent for inclusion before they participated in the study. The scope of the study is in line with Regulation (EC) No 1223/2009 of the European Parliament and Council, Cosmetics Europe–The Personal Care Association Guidelines “Product Test Guidelines for the Assessment of Human Skin Compatibility 1997”, Cosmetics Europe–The Personal Care Association “Guidelines for the Evaluation of the Efficacy of Cosmetic Products 2008”, and the World Medical Association (WMA) Declaration of Helsinki’s Ethical Principles for Medical Research Involving Human Subjects.

#### 2.3.5. Consumer Evaluation of Sensory Appeal

A preliminary consumer evaluation of the sensory appeal of the formulated cream prototypes was carried out, and a range of sensory parameters was determined, including skin smoothing, spreadability, pillow effect, homogeneity, consistency, adhesion, absorption, greasiness, and stickiness. The analysis was conducted using the scaling method in a group of 10 testers (women between the ages of 25 and 45) previously trained. Participants gave written informed consent to participate in the study. Each parameter was rated on a 5-point scale, where 1 point represents the lowest and 5 points the highest consumer assessment.

#### 2.3.6. Statistical Analyses

All statistical analyses were performed using the R package v. 3.6.1 [14]. All variables violated normality in at least one group (Shapiro–Wilk, *p* < 0.05). Therefore, non-parametric methods were used. The pooled Kruskal–Wallis test was followed by paired Wilcoxon tests with Holm correction (*n* = 5 per group). Statistical analyses were performed using ANOVA (StatSoft 13.1, Cracow, Poland).

## 3. Results and Discussion

### 3.1. Stability Analysis

**Figure 2:**
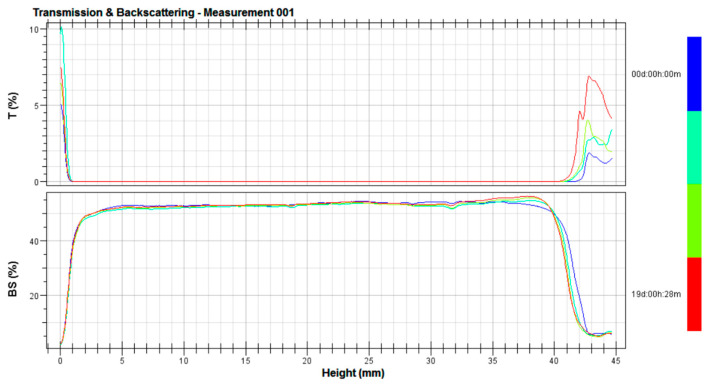
Example of the dependence of transmittance (T) and flux scattering (BS) on sample height for K4 face cream stored at 40 °C for 19 days; blue line—evaluation of the preparation immediately after application to the measuring vessel (time “0”), green line—evaluation after 7 days, yellow line—evaluation after 14 days, and red line—evaluation after 19 days.

Evaluation of the stability of cosmetic preparations is necessary to make sure that the product will retain its properties throughout its lifetime. It is important to exclude the probability of undesirable effects, causing a reduction in the quality of the preparation [15,16,17]. Evaluation of the stability of emulsions was carried out using Turbiscan, which allows characterization of physicochemical phenomena of a dispersion (such as particle migration, particle size change, flocculation, coalescence, sedimentation, creaminess, or aggregation) at a very early stage of its storage. The evaluation of prepared samples at the first stage was verified on the basis of transmittance (T) and backscattering (BS) dependence profiles. Based on the results obtained, it was clearly established that all the creams produced showed no signs of emulsion instability. Figure 2 shows an example of the backscattering (BS) and transmittance (T) dependencies as a function of cell height at a given time for an E4 face cream containing a mixture of both cellulose powders in a weight ratio of 2.5:2.5 with sizes of 2 and 7 µm, respectively, made from four measurements over 19 days.

**Figure 3:**
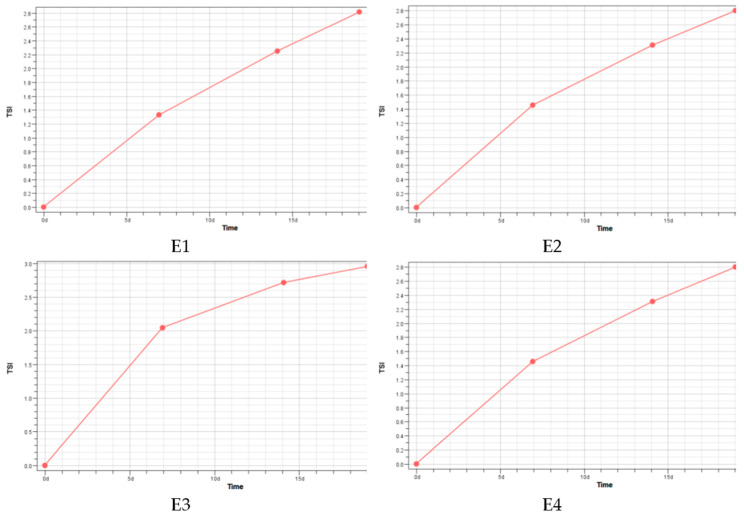
Turbiscan Stability Index (global) obtained for original face creams. E1-cream without spheroidal powders; E2-cream containing 5% *w*/*w* spheroidal cellulose powder of 2 µm; E3-cream containing 5% *w*/*w* spheroidal cellulose powder of 7 µm; E4-cream containing both spheroidal powders (2 and 7 µm) in a mass ratio of 2.5:2.5.

Another parameter carrying information about the stability of emulsion systems was the Turbiscan Stability Index (TSI), a dimensionless global destabilization parameter [18,19,20]. The Turbiscan Stability Index (Global TSI) corresponds to thetotal of all backscattering or transmission changes across the volume caused by destabilization. Therefore, the (Global TSI) of each emulsion was calculated at the end of the experiment as the average value of each TSI at the bottom, middle, and top of the measuring vessel [21]. The results obtained are shown in Figure 3.

Based on the test results obtained (Figure 3), it was concluded that the addition of spheroidal cellulose powders to emulsions does not adversely affect their stability. It should be noted that stabilization of emulsions with cellulose is hindered mainly due to its insolubility in water [22]. TSI values of a maximum of 3.0 have been obtained, whereby TSI can take values from 0 to 100.The higher the TSI value, the more unstable the sample is [23,24]. From the data presented, the results indicate the high stability of emulsions. In the literature, one can find examples of TSI studies for cosmetic emulsions with similar results [25].

### 3.2. Dynamic Viscosity

Rheology is an essential tool in the design and analysis of complex cosmetic formulations due to the inherent characteristics of their application. The viscosity of cosmetic emulsions is one of the most important characteristics evaluated by potential consumers. It influences the psychology of users during application, which can be a critical factor determining their impressions, ensuring that the cosmetic is easily picked up from the container and spread as a thin layer on the skin surface [26,27,28].

The literature contains reports on assessing the dynamic viscosity of various forms of cosmetic emulsions, including masks, creams, serums, or body lotions [29]. The form of a cosmetic significantly affects its viscosity. At the same time, dynamic viscosity values fall within a very wide range for one type of product. The dynamic viscosity of creams takes on values ranging from several to tens of thousands of mPa-s; the lighter the cream formulation, the lower the viscosity of the product [30,31].

**Figure 4:**
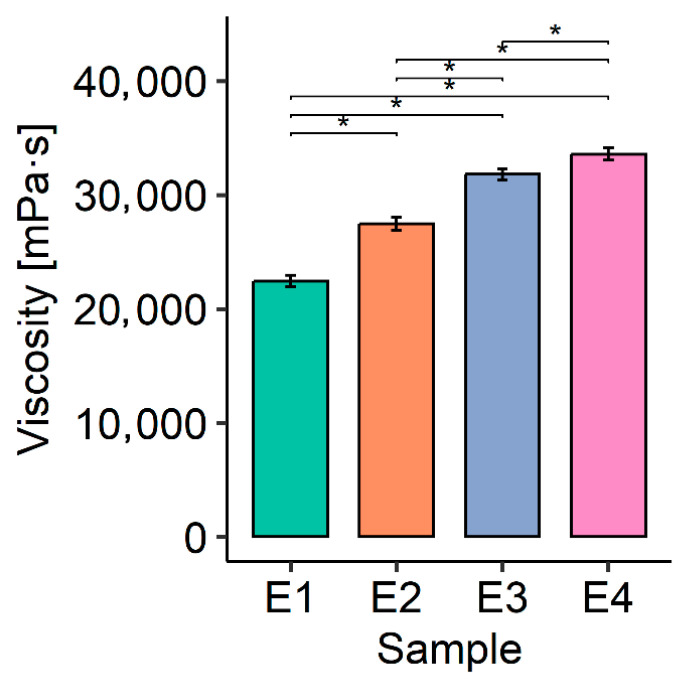
The influence of the content of spheroidal cellulose powders in tested facial creams on the values of their dynamic viscosity, *—significance level *p* < 0.05.

Viscosity rose step-wise with each compositional change (Table 3, Figure 4). The omnibus test confirmed a significant formulation effect (H = 17.9, *p* < 0.001). Holm-adjusted pair-wise Wilcoxon tests showed that every formulation differed from every other (all padj = 0.048). Between E1 and E4, the mean viscosity increased by 11.1 mPa·s (~50%).

The viscosity of the tested creams ranged from 22,440 to 33,575 mPa·s. The lowest viscosity (22,440 mPa·s) was obtained for the base cream (E1) without the addition of spheroidal cellulose powders. On the other hand, emulsion E4, containing a mixture of both cellulose powders in a weight ratio of 2.5:2.5 with sizes of 2 and 7 µm, respectively, had the highest viscosity (33,575 mPa·s). Based on the results, it can be concluded that the spheroidal cellulose powders contribute to the dynamic viscosity. In addition, the effect of the particle size of the spheroidal cellulose powders on emulsion viscosity can also be observed. Cream E2, which contains particles of 2 µm in size, obtained a lower viscosity (27,470 mPa·s) compared to that of cream E3 (31,767 mPa·s), which has particles of 7 µm in size in its composition.

The role of polymers as an example of rheology modifiers used in emulsion-based cosmetic formulations was presented by Różanska et al. [32]. In their article, they presented the results of a study of the rheological properties and stability of oil-in-water emulsions containing cellulose derivatives: methylcellulose, hydroxyethylcellulose, and hydroxypropylmethylcellulose. They found that fluids with high viscoelasticity were obtained, and the addition of methyl cellulose and hydroxypropyl methyl cellulose additionally led to highly flocculent emulsions. On the other hand, Martins et al. [33] prepared cosmetic creams (oil-in-water emulsions) using dry bacterial cellulose and carboxymethyl cellulose to investigate the possibility of partially or completely replacing surfactants while providing long-term stability and the required organoleptic properties. They found that bacterial cellulose and carboxymethyl cellulose provided very effective emulsion stabilization through the Pickering effect and continuous phase structuring. On the other hand, Dong et al [34] proved that the use of cellulose nanocrystals with a diameter of 30–60 nm in cosmetic emulsions resulted in stable systems with ultra-low viscosities, even around 50 mPa·s.

### 3.3. Texturometric Evaluation

The quality of cosmetic products, in the current era of the cosmetic industry, is becoming increasingly important. The texture of the product and the way it feels on the skin contribute greatly to the overall perception of product quality while also ensuring user satisfaction [35]. The author’s emulsions were subjected to textrometric evaluation, taking into account two parameters: hardness and adhesion strength. Hardness is the maximum force recorded during 1 test cycle. Adhesion force is the lowest (negative) value recorded during the test, which is a measure of the adhesion of the sample to the probe [27,36]. With the help of instrumental methods (textrometry), it is possible to obtain information about the mechanical, geometric, and surface attributes of a cosmetic product, which affect its perception by the user and its application functionality [37].

**Figure 5:**
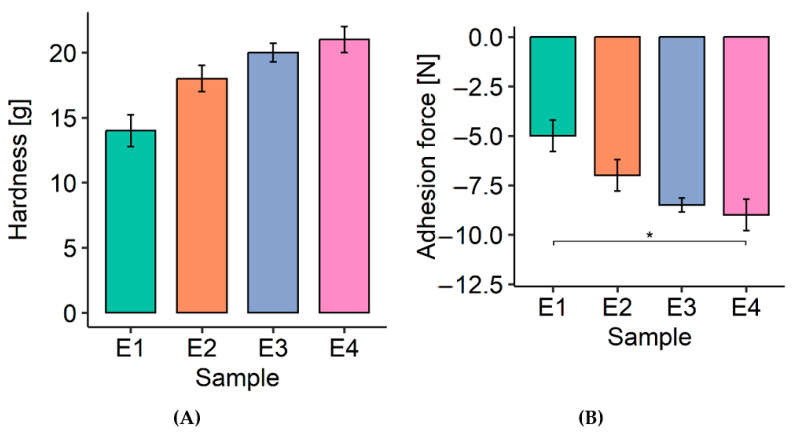
The impact of spherical cellulose powders on the values of hardness (**A**) and adhesion force (**B**) of face creams, *—significance level *p* < 0.05.

The results obtained in the texture analysis for the face creams are shown in Table 4 and Table 5 and Figure 5. Hardness displayed the same upward drift, but none of the six pair-wise contrasts survived Holm correction (all padj ≥ 0.064) (Table 4, Figure 5A). The 7 g rise from E1 to E4 is therefore suggestive but not definitive.

The overall effect of the formulation on adhesion was significant (Table 5, Figure 5B). Pair-wise analysis isolated a single robust difference—E1 vs. E4 (padj = 0.048)—an ~80% increase in pull-off force. All intermediate contrasts did not reach significance.

The addition of spheroidal cellulose powders to creams contributes to an increase in hardness values (5A) and a decrease in adhesion strength values (5B) compared to the base formulation. Creams containing spheroidal cellulose powders were characterized by higher values of the parameter compared to the base cream. The hardness values for the tested creams oscillated between 14 and 21 g, while the adhesion strength values ranged from −9 to −5 g. At the same time, the highest hardness value (21 g) and the lowest adhesion force value (−9) were possessed by cream E4, which contained Chroma Pur CV2 and CV7 spheroidal cellulose powders in a ratio of 2.5:2.5.The results obtained are coherent with the results obtained in the dynamic viscosity study. A texture analysis of cosmetic emulsions with polymers was presented by Gilbert et al. [38]. They observed that emulsions with polysaccharides (carob and xanthan) were characterized by hardness values in the range of 4 g to 6 g, and the adhesion force values measured for the tested creams were in the range of −1.5–2.5 g. On the other hand, in the work of Zięba et al. [17], the authors studied, among other things, the hardness and adhesion strength of emulsions with burdock powder and black turnip. The hardness value was in the range of 11.76−14.80 g, and the adhesion force was in the range of −10 to −5 g.

In conclusion, it was noted that the results obtained are within the range of results presented in the literature for cosmetic emulsions. They also allow us to conclude that the developed face creams with spheroidal cellulose powders will exhibit proper adhesion to the skin surface.

### 3.4. Degree of Skin Hydration

The restoration of the skin’s normal hydrolipidic barrier is extremely important in skin care treatments. This process involves restoring the integrity of the stratum corneum, rebuilding intercellular lipids, and regulating the skin’s natural hydration and protection mechanisms [39]. Formulations of modern cosmetics are rich in moisturizing and lubricating ingredients. Such examples are humectants and emollients, widely described in the literature [40,41,42]. Among them are also polymers, both natural and synthetic, such as hyaluronic acid, xanthan gum, polyethylene glycol, hydroxyethyl cellulose, carboxymethyl cellulose, nanocellulose, gelatin, etc. [43,44,45,46,47].

**Figure 6:**
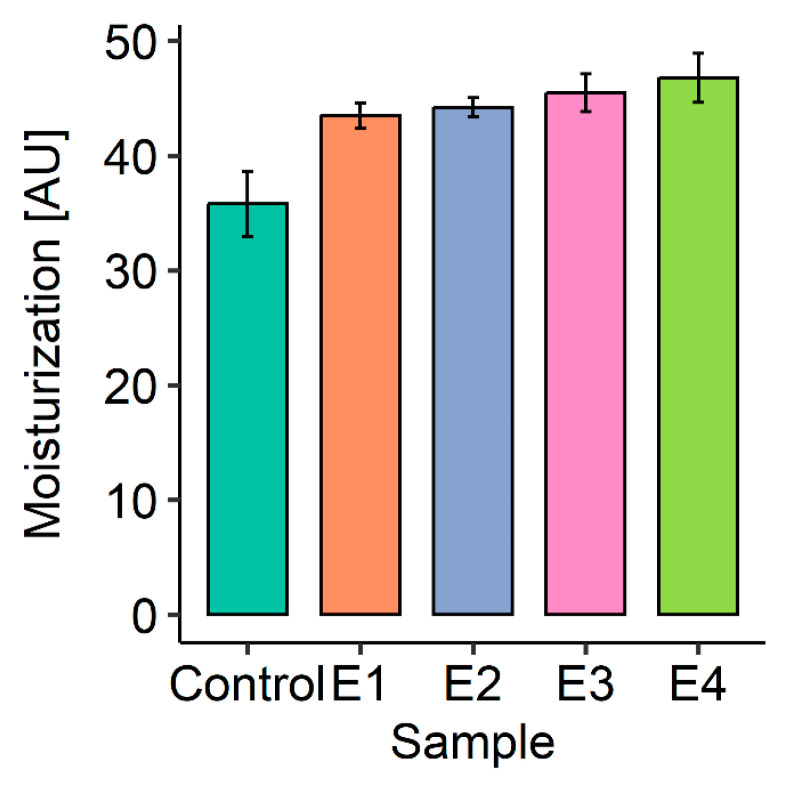
Comparison of skin hydration ability for the series of prototypes of facial creams including spherical cellulose powders.

The results obtained during corneometric measurements are shown in Table 6 and Figure 6. Moisturization climbed steadily (+31% from control to E4), but after Holm adjustment no pair-wise comparison crossed the 0.05 threshold (lowest padj = 0.079). The biological trend is evident, but statistical confirmation will require a larger sample or a less conservative correction.

The results obtained during corneometric measurements are shown in Figure 6.

The degree of hydration for the control area, i.e., the section of skin not treated with any cosmetic, was 35.8 a.u. Based on the results obtained, it can be observed that the application of each of the tested face creams caused an increase in the degree of skin hydration. The values of the evaluated parameter oscillate from about 43.5 (the degree of hydration after the application of the base cream) to the range of 44.2–46.8 a.u. (after application of creams with spheroidal cellulose powders). The highest skin hydration (46.8 a.u.) was observed after application of E4 cream, which contained both spheroidal cellulose powders differing in particle size (2 µm and 7 µm) introduced into the emulsion in a mass ratio of 2.5:2.5.

Moisturization of the skin after application of cosmetic emulsions was presented, among others, in their work by Roso et al. [48]. The effects were observed in a wide variety of volunteers living in four countries with different lifestyles, climates, and geographic environments (Brazil, France, India, and Mauritius). The results ranged from 25.0 to 44.2 a.u., taking into account the time from 0 to 24 h after emulsion application. Similar results of skin hydration after cosmetic application were obtained by Lotos et al. [49] for emulsions with xanthan gum and Daucus carota macerated oil. Lee et al. [50], on the other hand, developed a moisturizing nanocellulose-based composite by mixing TEMPO-oxidized cellulose nanofibers (TOCN) with hyaluronic acid (HA) and poly-γ-glutamic acid (γ-PGA). They demonstrated that the blend system can increase skin hydration through both physical moisture retention and biological mechanisms and can be used as an effective moisturizer in cosmetic applications.

Thus, in conclusion, it can be said that the obtained results of skin moisturization after application of creams with spheroidal cellulose powders are within the range of results presented in the literature. The conducted studies indicate that spheroidal cellulose powders combine favorable physicochemical properties with an effective mechanism of action that promotes skin hydration. It is important to remember that proper skin hydration plays a key role in maintaining the structural integrity of the stratum corneum, providing an optimal environment for enzymatic activity, regulating transepidermal water loss (TEWL), and in the functioning of defense mechanisms against pathogenic and physicochemical factors. Given the growing interest in natural and biocompatible with the environment ingredients, spheroidal cellulose powder can be an innovative functional additive in modern cosmetic formulations [51,52].

### 3.5. Consumer Evaluation of Sensory Appeal

Product aesthetics and sensory impressions play an important role in the perception and acceptance of cosmetic products. There are numerous scientific reports in the literature using sensory testing to evaluate cosmetic products [8,53,54]. Emulsion cosmetics come in the form of creams, lotions, gels, or foams and are the most commonly used body care products to improve the condition and feel of the skin. Achieving an optimal balance, when designing an emulsion formulation, between performance, efficacy, and sensory profile is highly anticipated. The sensory profile is a key factor influencing consumer satisfaction and the success of cosmetic products in the market [53,54,55]. When developing formulations of facial care emulsions, the main focus is on the consistency of the product, i.e., the use of rheology modifiers and consistency agents [56,57].

**Figure 7:**
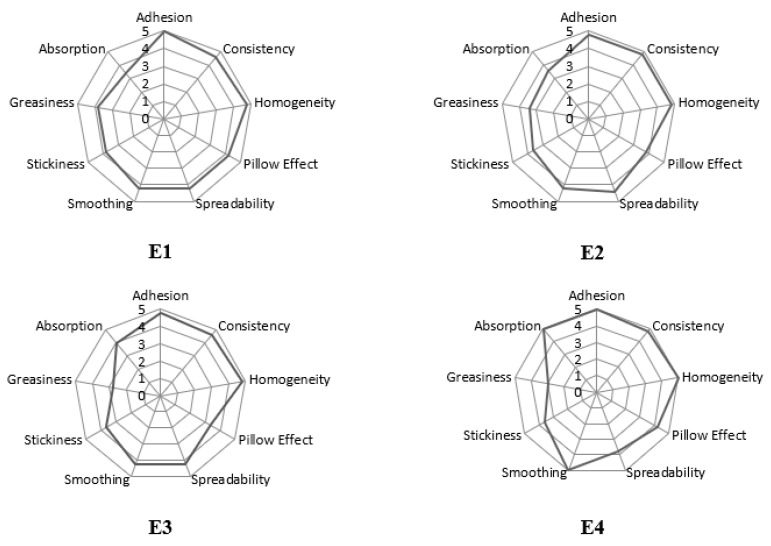
Consumer evaluation of sensory appeal of series of face creams containing spheroidal cellulose powders (CV2&CV7).

In this study, a preliminary consumer evaluation of the sensory appeal of facial creams with spheroidal cellulose powders was conducted. The following characteristics were evaluated: skin smoothing, spreadability, pillow effect, homogeneity, consistency, adhesion, absorption, greasiness, and stickiness. The obtained values of the sensory parameters are shown in graphs (Figure 7) of the so-called sensory profiles.

For the E1 base cream without the addition of cellulose powders, parameters such as adhesion, consistency, and homogeneity were rated the best (from 4.6 to 5 points) by the probands, and absorption and greasiness were rated the worst (from 2.8 to 3.4 points). E2 cream containing cellulose powder with a particle size of 2 µm was rated similarly. The parameters that received the highest ratings (4.8 to 5 points) were also adhesion, consistency, and homogeneity, while the lowest ratings were absorption, stickiness, and greasiness (2.8 to 3.6 points). Cream E3 with spheroidal cellulose powder CV7 received the highest ratings for the parameters of adhesion, consistency, and uniformity (from 4.6 to 4.8 points); the parameters rated worst for this cream were the pillow effect and greasiness (from 2.8 to 3.4 points). For the E4 cream, which contains both types of cellulose powders with particle sizes of 2 µm and 7 µm, the highest ratings were given to the following parameters: adhesion, smoothing, absorption, and homogeneity (maximum score—5 points), while the worst-rated parameters were spreadability, stickiness, and greasiness (from 3 to 3.6 points).

In summary, according to the members of the sensory panel, the emulsion E1, without spheroidal cellulose powders, was rated the lowest (total score of 34), while the face cream E4, which contains a mixture of spheroidal cellulose powders CV2&CV7, was rated the best. Thus, spheroidal cellulose powders were found to have a positive effect on face creams, showing exceptional sensory benefits. Roso et al. [58], in their work, also confirm the importance of functional components (texturizing/stabilizing polymer and emollient) on visual observation results and post-application sensation. On the other hand, Meneguello et al. [59] showed that sensory evaluations of emulsions containing balanced homopolymers underscored positive user acceptance, with participants preferring the skin feel and handling properties of these emulsions over synthetic alternatives.

## 4. Conclusions

Given the obvious and alarming effects of the use of microplastics on the ecosystem in many areas of life, including the cosmetics industry, it has become necessary to look for new solutions. One such example could be the use of natural, biodegradable cosmetic raw materials that will not adversely affect the physicochemical and functional properties of the final product.

The authors of this paper presented formulation solutions for the use of spheroidal cellulose powders in facial creams, which can be an alternative to microplastics. When designing facial formulations, it is necessary to consider both physicochemical and functional and sensory properties. Although efficacy and safety are considered the most important features of a product, application properties (viscosity, hardness, adhesion force, and sensory parameters) are key factors influencing consumer satisfaction when using modern cosmetic products. The presented results of the study confirm that the use of spheroidal cellulose powders makes it possible to obtain face creams in the form of emulsions that offer optimal structural integrality and high sensory attractiveness while maintaining the stability of the system over time. It should be noted that after the application of emulsions with spheroidal cellulose powders to the skin, there was also an increase in skin hydration compared to the control area.

In conclusion, it can be said that spheroidal cellulose powders can be an interesting alternative to cosmetic products containing microplastics. Since spheroidal cellulose powders used in facial creams have been found to have favorable physicochemical and functional properties, they will be the subject of further research. The authors plan to extend the research toward demonstrating their applicability as potential anti-aging ingredients through diagnostic studies of skin conditions.

## Figures and Tables

**Table 1:** Particle characterization [7].

Particle Characterization	Cellulose 2 µm	Cellulose 7 µm
Buk density	0.30 g/cm^3^	0.56 g/cm^3^
Surface area	3.5 m^2^/g	1.0 m^2^/g
Refractive index	1.54	1.54
Oil up-take	46.1 g/100 g	44.6 g/100 g

**Table 2:** Formulations of the original face creams.

Phase	INCI Name	Concentration [% *w*/*w*]			
		E1	E2	E3	E4
I	*Hedera Helix (Ivy) Leaf Extract*	3.0			
	Cetearyl Alcohol	3.0			
	Cetearyl Olivate and Sorbitan Olivate	5.0			
II	Aqua	to 100			
	Cellulose (CV 2)	-	5.0	-	2.5
	Cellulose (CV 7)	-	-	5.0	2.5
III	Tocopheryl Acetate	0.5			
	Sodium Benzoate and Potassium Sorbate	0.5			

**Table 3:** Mean, standard deviation, median, and Kruskal–Wallis statistics for the viscosity analysis of the study samples.

Sample	*n*	Mean ± SD (mPa·s)	Median (mPa·s)
E1	5	22,440 ± 508	22,500
E2	5	27,470 ± 561	27,300
E3	5	31,807 ± 490	31,800
E4	5	33,575 ± 534	33,700
Kruskal–WallisH		17.86	*p* = 4.7 × 10^−4^

**Table 4:** Mean, standard deviation, median, and Kruskal–Wallis statistics for the hardness analysis of the study samples.

Sample	*n*	Mean ± SD (g)	Median (g)
E1	5	14.0 ± 1.22	14.0
E2	5	18.0 ± 1.00	18.0
E3	5	20.0 ± 0.71	20.0
E4	5	21.0 ± 1.00	21.0
Kruskal–Wallis H		16.66	*p* = 8.3 × 10^−4^

**Table 5:** Mean, standard deviation, median, and Kruskal–Wallis statistics for the adhesion force analysis of the study samples.

Sample	*n*	Mean ± SD (g)	Median (g)
E1	5	−5.0 ± 0.79	−5.0
E2	5	−7.0 ± 0.79	−7.0
E3	5	−8.5 ± 0.35	−8.5
E4	5	−9.0 ± 0.79	−9.0
Kruskal–WallisH		15.98	*p* = 1.1 × 10^−3^

**Table 6:** Mean, standard deviation, median, and Kruskal–Wallis statistics of skin moisturization analysis of study samples.

Sample	*n*	Mean ± SD (AU)	Median (AU)
Control	5	35.8 ± 2.84	35.2
E1	5	43.5 ± 1.07	43.9
E2	5	44.2 ± 0.85	44.4
E3	5	45.5 ± 1.65	45.4
E4	5	46.8 ± 2.14	46.6
Kruskal–WallisH		18.04	*p* = 1.2 × 10^−3^

## Data Availability

The original contributions presented in this study are included in the article. Further inquiries can be directed to the corresponding author.
